# Biophysical characterization of chloride intracellular channel 6 (CLIC6)

**DOI:** 10.1016/j.jbc.2023.105349

**Published:** 2023-10-12

**Authors:** Veronica Loyo-Celis, Devendra Patel, Shridhar Sanghvi, Kamalpreet Kaur, Devasena Ponnalagu, Yang Zheng, Sahej Bindra, Harmeet Rireika Bhachu, Isabelle Deschenes, Shubha Gururaja Rao, Harpreet Singh

**Affiliations:** 1 Department of Physiology and Cell Biology, College of Medicine, The Ohio State University, Columbus, Ohio, USA; 2 Department of Molecular Cellular and Developmental Biology, The Ohio State University, Columbus, Ohio, USA; 3 Department of Pharmacology, The University of Washington, Seattle, Washington, USA; 4 Raabe College of Pharmacy, Ohio Northern University, Ada, Ohio, USA

**Keywords:** chloride channel, redox-regulation, pH-regulation, IAA-94, anion transport

## Abstract

Chloride intracellular channels (CLICs) are a family of proteins that exist in soluble and transmembrane forms. The newest discovered member of the family CLIC6 is implicated in breast, ovarian, lung gastric, and pancreatic cancers and is also known to interact with dopamine-(D(2)-like) receptors. The soluble structure of the channel has been resolved, but the exact physiological role of CLIC6, biophysical characterization, and the membrane structure remain unknown. Here, we aimed to characterize the biophysical properties of this channel using a patch-clamp approach. To determine the biophysical properties of CLIC6, we expressed CLIC6 in HEK-293 cells. On ectopic expression, CLIC6 localizes to the plasma membrane of HEK-293 cells. We established the biophysical properties of CLIC6 by using electrophysiological approaches. Using various anions and potassium (K^+^) solutions, we determined that CLIC6 is more permeable to chloride-(Cl^−^) as compared to bromide-(Br^−^), fluoride-(F^−^), and K^+^ ions. In the whole-cell configuration, the CLIC6 currents were inhibited after the addition of 10 μM of IAA-94 (CLIC-specific blocker). CLIC6 was also found to be regulated by pH and redox potential. We demonstrate that the histidine residue at 648 (H648) in the C terminus and cysteine residue in the N terminus (C487) are directly involved in the pH-induced conformational change and redox regulation of CLIC6, respectively. Using qRT-PCR, we identified that CLIC6 is most abundant in the lung and brain, and we recorded the CLIC6 current in mouse lung epithelial cells. Overall, we have determined the biophysical properties of CLIC6 and established it as a Cl^−^ channel.

The chloride channels are present in all the cellular membranes and are tightly regulated by various stimuli including voltage, pH, volume, ligands, pressure, and intracellular messengers (1, 2). These channels are preferentially permeable to chloride and often allow other smaller anions as well as cations to pass through them (3). In cells, physiologically chloride channels play a vital role in regulating their homeostasis, including stabilization of cell membrane potential, transepithelial transport, maintenance of intracellular pH, cell proliferation, fluid secretion, and regulation of cell volume (2, 4). Several families of chloride channels have been identified over the past 3 decades. Members of the recently characterized chloride intracellular channel (CLIC) protein family have been reported in intracellular organelles (5). CLICs have six paralogs, CLIC1-6, and they exist in soluble as well as membrane forms (6). Their dysregulation is associated with cancer (7, 8), Alzheimer’s (9), pulmonary hypertension (10), hearing loss (11), angiogenesis (12), obesity (13), and cardioprotection (14, 15).

Intracellular chloride channels are ubiquitously present in many organellar membranes where they are specifically involved in the regulation of pH, volume, and ionic homeostasis of individual organelles (4). CLIC family members are found in the nucleus (16), mitochondrial (14, 15, 17), endoplasmic reticulum (18), Golgi apparatus (19), and lysosomal membranes (20) and as a soluble protein in the cytosol (21). CLIC6, the most recent CLIC family member was originally identified in rabbit gastric parietal cells and named parchorin (5, 22, 23, 24, 25, 26). CLIC6 is present in the conserved gene cluster ACD (AML/CLIC/DSCR1-like) in chromosome 21 and is the longest known isoform of CLIC proteins (23, 25, 26). Other CLIC proteins present in the ACD gene cluster are CLIC4 (chromosome 4) and CLIC5 (chromosome 6), which also indicate that these channels might have overlapping distribution and functional properties in cells. CLIC6 shares the structural homology with other CLIC proteins as well as the glutathione s-transferase superfamily (5, 27). Though the gene was discovered over 2 decades ago (23, 24), evidence of the ability of CLIC6 to form an ion channel is still lacking. A genome-wide association study recently associated CLIC6 with psoriasis (28), lung function (29), accelerated aging associated with alcohol use (30), and opioid targets in cancer treatment (31).

CLIC6 also known as parchorin when overexpressed in the LLC-PK1 kidney cell line potentiated the efflux of Cl^−^ from cells. The Cl^−^ flux was observed only when Cl^−^ was depleted from the extracellular solution implicating CLIC6 in cellular Cl^−^ transportation (26). In contrast, cotransfection of CLIC6 with dopamine D3-receptors in CHO cell lines fails to present any CLIC6-mediated Cl^−^ fluxes raising concerns about the ability of CLIC6 to form an ion channel (24). However, in both cell systems, ectopic overexpression forced CLIC6 to localize to the plasma membrane of CHO and LLC-PK1 cells, and Cl^−^ flux measurements were done by Cl^−^ sensors such as MQAE and SPQ, respectively (24, 26). In this study, we set out to characterize CLIC6 and its ability to form ion channels in cell membranes. Since CLIC proteins are known to form ion channels on ectopic expression in cell lines (32, 33) and planar bilayers (27, 34, 35, 36, 37), we probed for evidence for CLIC6 to form an ion channel. On ectopic expression in HEK-293 cells, we found that CLIC6 is an anion channel that is sensitive to pH and redox regulation. Our qPCR analysis demonstrated that CLIC6 is abundant in lung and brain tissues as compared to other organs. Accordingly, we describe here the properties of CLIC6 in mouse lung epithelial (MLE) cells. Our data for the first time show that CLIC6 forms a functional ion channel that preferentially allows chloride over other anions.

## Results

### Characterization of CLIC6

**Figure 1:**
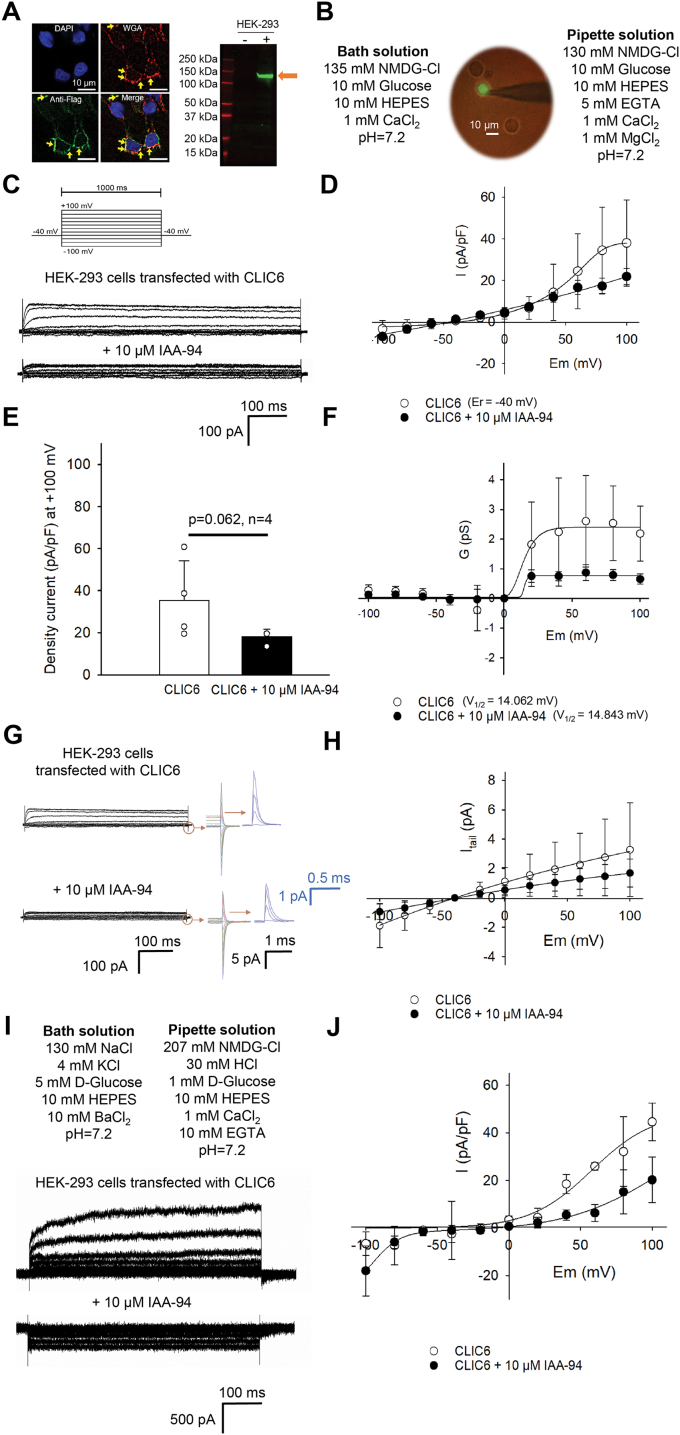
**Characterization of CLIC6 in HEK-293 cells.***A*, HEK-293 cells transfected with CLIC6 were labeled with DAPI (*blue*, nucleus marker), wheat germ agglutinin (*red*, plasma membrane marker), and anti-FLAG (for FLAG-CLIC6, *green*, 1:500, Sigma Aldrich (F1804)). Anti-mouse Alexa 488 was used to label CLIC6 protein. Merge show overlay of DAPI-WGA and anti-FLAG fluorescent images. *Yellow arrows* indicate the expression of FLAG-CLIC6 at the plasma membrane in transfected cells. Bar scale:10 μm. Western blot with anti-FLAG [1:500, Sigma Aldrich (F1804)] showing FLAG-tagged CLIC6 in transfected HEK-293 cells. The *orange arrow* indicates FLAG-CLIC6 migrates at ∼120 kDa. *B*, fluorescent image showing transfected cells (CLIC6 co-transfected with GFP, *green*) attached to a patch pipette. The composition of the bath (extracellular) and pipette (intracellular) solution is given. *C*, biophysical and pharmacological characterization of the CLIC6 current. Voltage pulses were applied from −100 to +100 mV in 20 mV increments (holding potential of −40 mV, step duration 1000 ms). Representative trace of whole-cell recordings of CLIC6 expressed in HEK-293 cells before (*top*) and after adding 10 μM of IAA-94 (*bottom*). *D*, voltage-dependence of CLIC6. Current-voltage (I-V) plot of the recordings is shown in *C*. The CLIC6 current was measured at 40 ms after voltage steps are applied and normalized to cell capacitance. Current–voltage plot after the addition of 10 μM of IAA-94 (*black circle*). The reversal potential calculated from the I-V plot was −40 mV. Cl^−^ currents saturate at higher voltages (≥80 mV). *E*, bar graph representing peak amplitude at +100 mV for CLIC6 in the absence (*white*) and presence (*black*) of 10 μM IAA-94. IAA-94 blocked a large proportion of the CLIC6 currents (*p* = 0.062, n = 4). *F*, G–V plot for CLIC6. The conductance G [calculated from I_Cl_/(E-E_Cl_)] was plotted as a function of voltage. Solid line fit to the Boltzmann function. *G*, the tail currents of CLIC6 (in different colors) in enhanced detail from −100 mV to +100 mV before and after the addition of 10 μM IAA-94. Blue traces are the tail current from +40 to +100 mV. *H*, the tail current as a function of membrane potential was obtained by analyzing the tail current peak at 1.025s (time in which the maximum tail current is without having contamination with the capacitive component) before and after the addition of 10 μM IAA-94. Solid line fit to the Boltzmann function. *I*, composition of extracellular and intracellular solutions used in electrophysiological recordings with SyncroPatch 384i. Representative trace of CLIC6 expressed in HEK-293 cells recorded in whole-cell configuration with automated patch clamp system. *J*, current–voltage curve obtained for 13 cells recorded from automated patch clamp system. Error bars represent the mean ± standard deviation (SD), and significance was calculated by student’s *t* test (paired). CLIC, Chloride intracellular channel.

As an ectopic expression of CLIC6 in cell lines result in its localization to the plasma membrane (24, 25), we overexpressed CLIC6 in HEK-293 cells (Fig. 1). CLIC6 was cloned with an N terminus Flag tag, and the expression of the protein was verified in HEK-293 cells by Western blot (Fig. 1*A*). Transfected HEK-293 cells were also probed with anti-FLAG antibodies. As shown in Figure 1*A*, upon ectopic expression in HEK-293 cells, CLIC6 is expressed near the plasma membrane where it colocalizes with the wheat germ agglutinin indicating its possible presence near the plasma membrane. To measure the channel activity, we performed a whole-cell patch-clamp on HEK-293 cells transfected with CLIC6 and GFP in NMDG-Cl solutions (Fig. 1, *B*–*I*). We observed a large current at positive holding potentials (Fig. 1, *C* and *D*). CLIC6 in HEK-293 cells exhibits fast gating, which is voltage-dependent (V_1/2_ = 14.062 mV). The fast gate closes at negative membrane voltages and opens upon depolarization to positive voltages (Fig. 1, *C* and *D*). The reversal potential (Er) for CLIC6 was −40 mV, the same value as the holding potential used in the voltage step protocol that is consistent with the predicted reversal potential to chloride (−0.6 mV) calculated in the ion conditions described in Figure 1*B*.

IAA-94 is a known blocker of CLIC proteins (27, 38, 39). The analysis of the instantaneous current amplitude at the different voltage steps indicated the presence of a constitutive Cl^−^ selective current, which probably originated from CLIC6 as IAA-94 blocked these currents (Fig. 1*D*). Addition of 10 μM IAA-94 in bath solution led to a significant block at positive holding potentials (Fig. 1, *D* and *F*). The peak current at +100 mV was blocked by 48 ± 5% on the addition of IAA-94 (Fig. 1*E*). Surprisingly, we did not observe any block of CLIC6 on negative potentials. In parallel, nontransfected HEK-293 cells lacking CLIC6 were also obtained under the same recording conditions (Fig. S1). Although Cl^−^ currents were present in nontransfected HEK-293 cells, they were not blocked by IAA-94 (Fig. S1, *C* and *D*). To determine the voltage dependence of the CLIC6 currents, the conductance (G) was plotted as a function of the voltage and fitted to the Boltzmann equation (Fig. 1*F*). CLIC6 shows enhanced activity on positive holding potentials as compared to the negative holding potentials as shown by G/V graph (Fig. 1*F*). To confirm the voltage dependency at positive membrane potentials, voltage steps ranging from −100 to +100 mV were applied followed by a voltage step to −40 mV to elicit tail currents (Figs. 1*G*, and S2). The tail current as a function of membrane potential was obtained by measuring the peak tail current (at 1.025 s) before and after the addition of 10 μM IAA-94.

Independent of the traditional patch-clamp approach, we also probed HEK-293 cells transfected with CLIC6 by using an automated patch-clamp approach with SyncroPatch 384i (Nanion technologies (Fig. 1*I*). Each well of the SyncroPatch 384i has one aperture which is connected to an individual head stage of the amplifier. Hence, each well is designated as an independent experiment (n = 1). With PatchControl 384i, all the parameters including seal resistance, capacitance, and series resistance were determined from individual wells after the application of a test pulse. All parameters are monitored in real-time and can be recorded for individual experiments for each electrode. We successfully recorded currents from 13 individual HEK-293 cells transfected with CLIC6 (Fig. 1*I*). We obtained the current–voltage (I-V) relationship using a step protocol (Fig. 1*C*). The whole-cell currents presented rectification at positive holding potentials (Fig. 1*J*) similar to traditional whole-cell approach (Fig. 1*D*).

**Figure 2:**
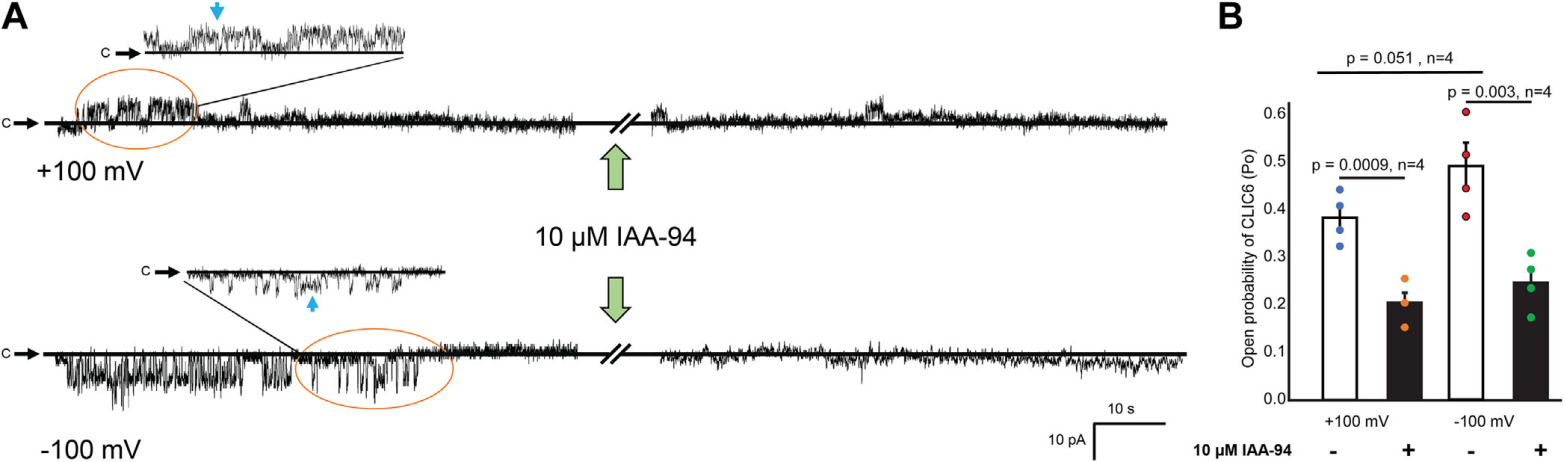
**Single-channel current is blocked by IAA-94.***A*, single-channel recordings under control conditions (135/130 mM NMDG-Cl; *cis*/*trans*) at +100 mV (*top*) and −100 mV (*bottom*) and immediately after the addition of 10 μM IAA-94 in the bath solution. The *green arrow* indicates the addition of IAA-94, and *solid black lines* represent the closed levels (c). Inset shows an enlarged image of CLIC6 recording for a short duration. *Blue arrows* indicate the substate level at 50% of the main opening. Note the reduction in the single-channel activity of CLIC6 after the addition of 10 μM IAA-94 at +100 mV and −100 mV. The data were filtered at 20 kHz and sampled at 40 kHz. All the current traces in this figure are from the same patch. *B*, bar graph of the average of normalized CLIC6 open probability (Po) of CLIC6 at +100 mV and −100 mV (n = 4). IAA-94 significantly blocked the CLIC6-mediated Cl^−^ currents at +100 mV by 53 ± 4% (*p* = 0.0009, n = 4) and 51 ± 5% at −100 mV (*p* = 0.003, n = 4). The difference between Po at −100 and +100 mV in absence of IAA-94 was (*p* = 0.051, n = 4). Error bars represent the mean ± standard deviation (SD), and significance was calculated by Students’ *t* test (paired). CLIC, Chloride intracellular channel.

Next, we tested whether single-channel currents can also be blocked by IAA-94. We recorded CLIC6 single-channel activity in cell-attached configuration (where the chloride composition was 130 mM for the pipette and 4.2 mM for the cytoplasm) at +100 mV and −100 mV for 100 s and subsequently added 10 μM IAA-94 in the bath solution. In single-channel recordings, we noticed two distinct substates of the channel in addition to a large current (Fig. 2*A*). The Po of CLIC6 decreased by 53 ± 4% and 51 ± 5% at +100 mV and −100 mV, respectively, on the addition of 10 μM IAA-94 (n = 4, Fig. 2*B*). We also observed a substate (at 50% level of the main opening, *blue arrows* in Fig. 2*A*) which was also reported for other CLIC proteins (35). Our electrophysiology approaches conclusively demonstrate that CLIC6 can form a functional channel that can be blocked by IAA-94.

### CLIC6 forms a Cl-selective channel

**Figure 3:**
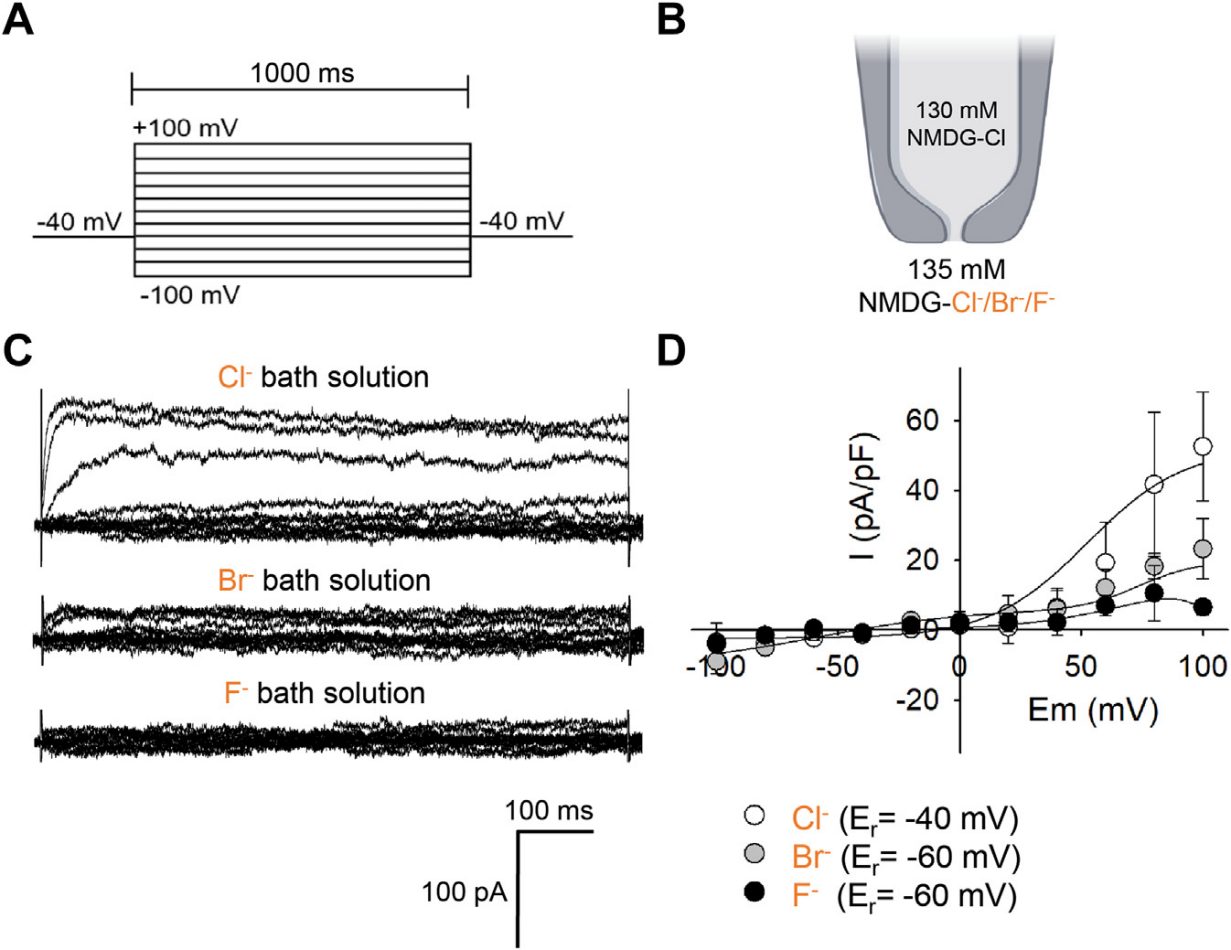
**CLIC6 forms a Cl-selective channel.** The effect of changing the external anion composition for CLIC6 was analyzed by whole-cell patch-clamp. *A*, voltage-step protocol. *B*, recording solutions were kept constant in the pipette, but external anions were replaced from Cl^−^ to Br^−^ or F^−^. *C*, representative whole-cell recordings for HEK-293 cells transfected with CLIC6 under different anions in the bath solution. *D*, voltage-dependence of the instantaneous current amplitudes normalized to HEK-293 cells capacitance in standard external solution (135 mM Cl^−^, *open circle*) and from the same cell after the extracellular solution has been changed to 135 mM Br^−^ (*gray circle*) and 135 mM F^−^ (*black circle*). Data were fit to the Boltzmann function and shown with a *solid line*. Permeability ratios were determined using data from D by solving the Goldman–Hodgkin–Katz equation (n = 5 independent experiments). CLIC, chloride intracellular channel.

CLIC proteins are known to form anion-selective channels. The selectivity of CLIC6 was measured by perfusing different anion or cation solutions in the bath. The pipette was kept at a constant 130 mM Cl^−^ concentration with the NMDG-Cl solution (Fig. 3*B*). With 135 mM NMDG-Cl in the bath solution and 130 mM NMDG-Cl in the pipette, we observed large currents in HEK-293 cells transfected with CLIC6. The channel was highly active on depolarizing positive voltages (Fig. 3, *C* and *D*). On replacing NMDG-Cl with 135 mM NMDG-Br or NMDG-F in bath solution in the same HEK-293 cell transfected with CLIC6, we observed a significant decrease in whole-cell currents (Fig. 3*C*). The reversal potential (E_r_) for Cl^−^ was −40 mV, and when Cl^−^ was replaced with Br^−^ and with F^−^, it was −60 mV (Fig. 3*D*). The activity of CLIC6 significantly dropped in Br^−^ and F^−^ solutions (Fig. 3*C*). In Br^−^, we observed a similar current kinetics at positive voltages as seen for Cl^−^ ions albeit with a small current amplitude (Fig. 3, *C* and *D*). There was hardly any activity observed in the solution containing F^−^ ions. Our results indicate that the selectivity of CLIC6 is Cl^−^ >> Br^−^ = F^−^. CLICs are known to form poorly selective ion channels. We also tested whether CLIC6 can allow potassium ions. The bath solution was replaced with KCl and potassium methyl sulfate (KMeSO_4_). Under KCl, a small current was observed in HEK-293 cells transfected with CLIC6 which was ablated in KMeSO_4_ (Fig. S3, *A* and *B*). Similar to NMDG-Cl, in KCl, CLIC6 was more active on positive voltages. In nontransfected HEK-293 cells, we did not observe larger currents as recorded in CLIC6-transfected cells when the bath solution was replaced with KCl or KMeSO_4_ (Fig. S3, *C* and *D*). These results suggest that CLIC6 is an IAA-94-sensitive Cl^−^ selective ion channel.

### pH regulation of CLIC6

**Figure 4:**
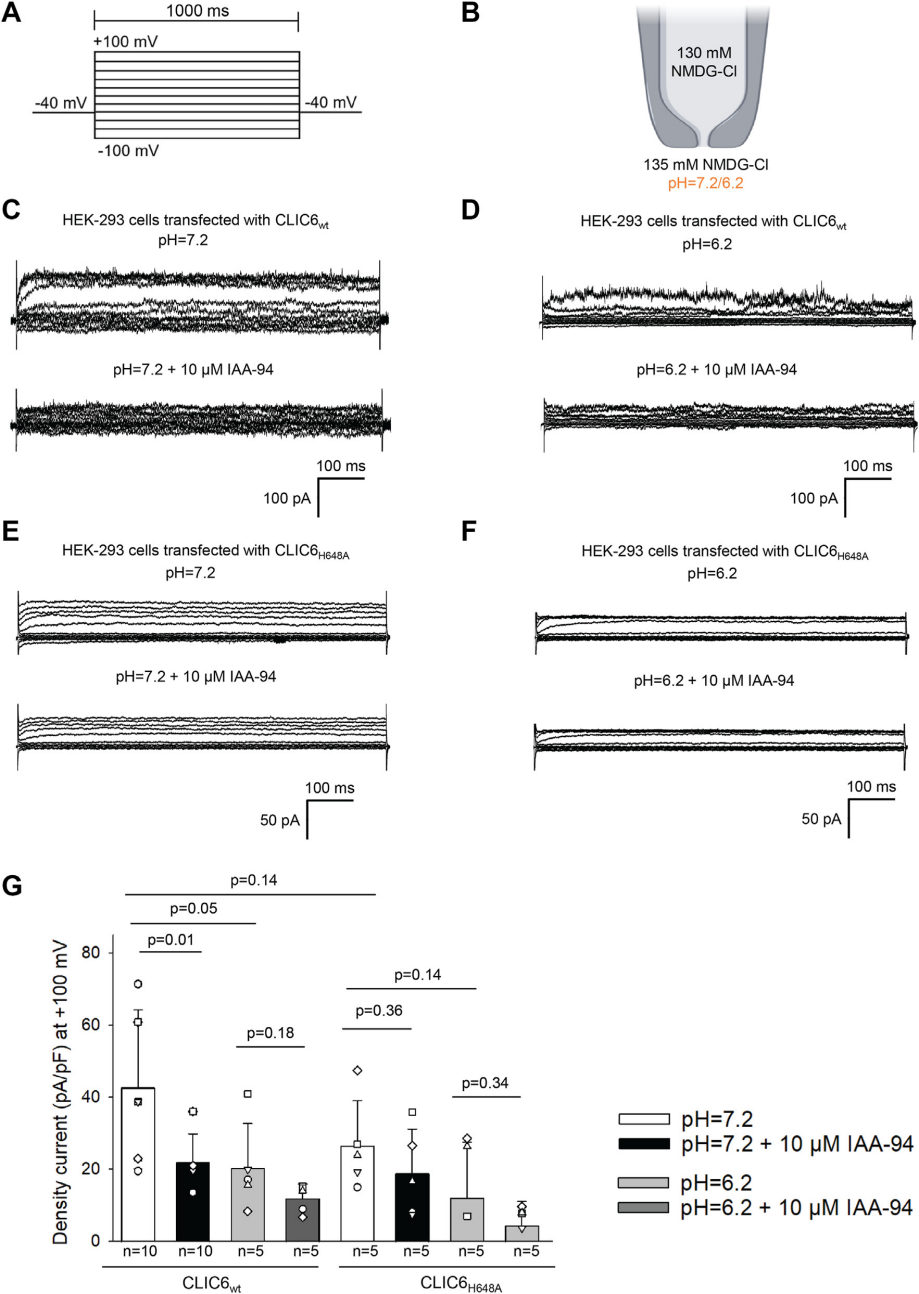
**Effect of pH on CLIC6 currents.** External acidification (pH 6.2) decreases rectifying chloride current in HEK-293 cells transfected with CLIC6. *A*, voltage-step protocol. *B*, recording solutions were kept constant in the pipette, but the pH was acidified from 7.2 to 6.2. *C*, representative trace from whole-cell patch-clamp recordings performed on HEK-293 cells transfected with CLIC6 and bathed in NMDG-Cl solution at pH 7.2 (*top*). The whole-cell currents were blocked after the addition of 10 μM IAA-94. *D*, lowering pH to 6.2 decreased Cl^−^ currents in HEK-293 cells transfected with CLIC6. The addition of 10 μM IAA-94 had no impact on the whole-cell current. *E*, the representative traces from HEK-293 cells transfected with the mutant CLIC6_H648A_ and bathed in NMDG-Cl solution at pH 7.2 before (*top*) and after the addition of 10 μM IAA-94 (*bottom*), there was no change in the whole-cell current. *F*, HEK-293 cells expression CLIC6_H648A_ at pH 6.2 showed no change in whole cell currents on the addition of 10 μM IAA-94. *G*, current density (pA/pF) bar graphs of HEK-293 cells transfected with CLIC6 and CLIC6_H648A_ before and after adding 10 μM IAA-94 at pH = 7.2 (*p* = 0.01, n = 10 and *p* = 0.36, n = 5 respectively) and 6.2 (*p* = 0.18, n = 5 and *p* = 0.34, n = 5, respectively). Error bars represent the mean ± standard deviation (SD), and significance was calculated by ANOVA (1-way). CLIC, chloride intracellular channel.

For CLIC proteins, an elegant model for the dual regulation of these channels by redox and pH has been proposed (35, 40, 41). Lower pH is well characterized for modulating anion fluxes across cellular membranes (42, 43). To analyze the impact of pH on CLIC6, we expressed CLIC6 in HEK-293 cells and carried out whole-cell patch-clamp recordings. On changing pH from 7.2 to 6.2, we observed a slight decrease but not a significant change in current density for CLIC6 (Fig. 4, *C*–*G*). In addition to 10 μM IAA-94 (Fig. 4, *C* and *G*), we observed a similar block of whole-cell currents in HEK-293 cells transfected with CLIC6 at pH 7.2 as observed in Figure 1, *C* and *D*. Surprisingly, the block was not significant at a lower pH 6.2 (Fig. 4, *D* and *G*) in HEK-293 cells transfected with CLIC6. Our experiments support a possibility (44) that histidine residues located in the C terminus are involved in coordinating pH-dependent conformational change. Two histidine residues in CLIC1 are shown to be involved in the pH-dependent conformational stability of wildtype CLIC1 *via* their protonation (44). In CLIC6, only histidine at position 648 is conserved which aligns with histidine 185 for CLIC1 (Fig. S4). We predict that H648 is directly involved in a pH-dependent conformational change in CLIC6. We mutated the H648 to alanine residue to test its role in the modulation of CLIC6 by pH. CLIC6 H648A mutant was transfected in HEK-293 cells, and currents were recorded at pH 7.2 (Fig. 4*E*) and pH 6.2 (Fig. 4*F*). CLIC6 H648A showed a significant reduction in the current density, but the difference between pH 7.2 and 6.2 was ablated (Fig. 4, *E*–*G*). We also observed that CLIC6 H648A was not blocked by IAA-94 (Fig. 4, *F* and *G*). Our results implicate that H648 is a key residue involved in the pH-mediated conformational change of CLIC6.

### Redox CLIC6 regulation

**Figure 5:**
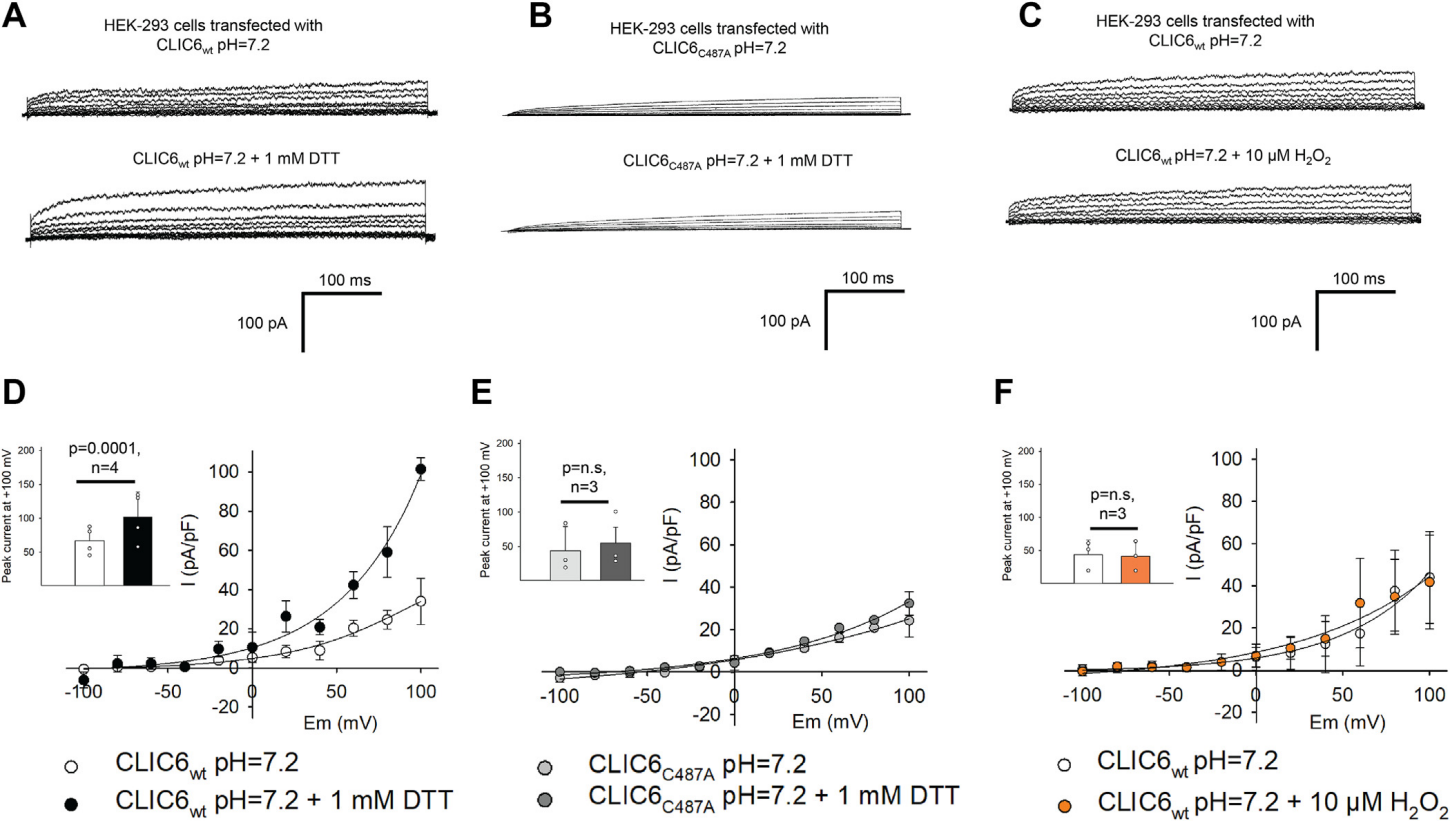
**DTT regulates the activity of CLIC6.** Effect of dithiothreitol (DTT) and hydrogen peroxide (H_2_O_2_) on the activity of CLIC6 in whole-cell patches in HEK-293 cells transfected with CLIC6 at pH 7.2. *A*, representative recordings of CLIC6 under control conditions and after addition of 1 mM DTT. *B*, representative recordings of CLIC6 C487A mutant under control conditions and after addition of 1 mM DTT. *C*, representative traces of CLIC6 under control conditions and after the addition of 10 μM H_2_O_2_. *D*, current–voltage relationship of CLIC6 under control and 1 mM DTT. Inset is a bar graph representing peak current at +100 mV. Significant difference from control after the addition of 1 mM DTT (*p* =0.0001, n = 4). *E*, current–voltage curve of CLIC6 C487A mutant (p = n.s, n = 3), under control and after the addition of 1 mM DTT. Inset showing a bar graph of peak current at +100 mV with and without DTT for CLIC6 C487A mutant. There was no impact of DTT on the activity of the CLIC6 C487A mutant. *F*, curve–voltage plots for CLIC6 with (*orange circle*) and without (white circle) 10 μM H_2_O_2_. Inset representing peak current at +100 mV for CLIC6 in the presence (*orange*) and absence (*white*) of 10 μM H_2_O_2_ (p = n.s, n = 3). Error bars represent the mean ± standard deviation (SD), and significance was calculated by Students’ *t* test (paired). CLIC, Chloride intracellular channel.

The activity of CLIC proteins is regulated by redox potential and specifically DTT is known to augment the channel function (35, 45, 46). We also tested whether DTT had any effect on the activity of CLIC6. In addition to 1 mM DTT in the bath solution, the peak current at positive holding potentials significantly increased (*p* = 0.0001, n = 4, Fig. 5, *A* and *D*). In a planar bilayer system, DTT promotes the insertion of the protein into the membrane (27, 35, 36, 37), but here we show that the activity of the inserted channel is affected by DTT as the impact was noticed within 100 milliseconds. Free thiol groups of cysteine residue at position 24 in CLIC1 are the major targets of redox regulation of CLIC1 activity (35). Therefore, we compared the number and location of cysteine residues within the transmembrane domain of CLIC1 and CLIC6 (Fig. S4). In CLIC6, cysteine at position 487 is a corresponding residue of C24 of CLIC1 (35) which suggests that this residue could be a possible redox sensor (Fig. S4). Mutation of C487 to alanine (C487A) significantly reduced the activity of CLIC6 as reported for CLIC1 (35). In parallel, when DTT was added to the CLIC6 C487A mutant, we did not notice any change in the activity of CLIC6 C487A (Fig. 5, *B* and *E*). The addition of 10 μM H_2_O_2_ in the bath solution had no impact on the activity of CLIC6, (Fig. 5, *C* and *F*). These results implicate C487 residue in the DTT-mediated regulation of CLIC6 activity.

### Distribution of CLIC6

**Figure 6:**
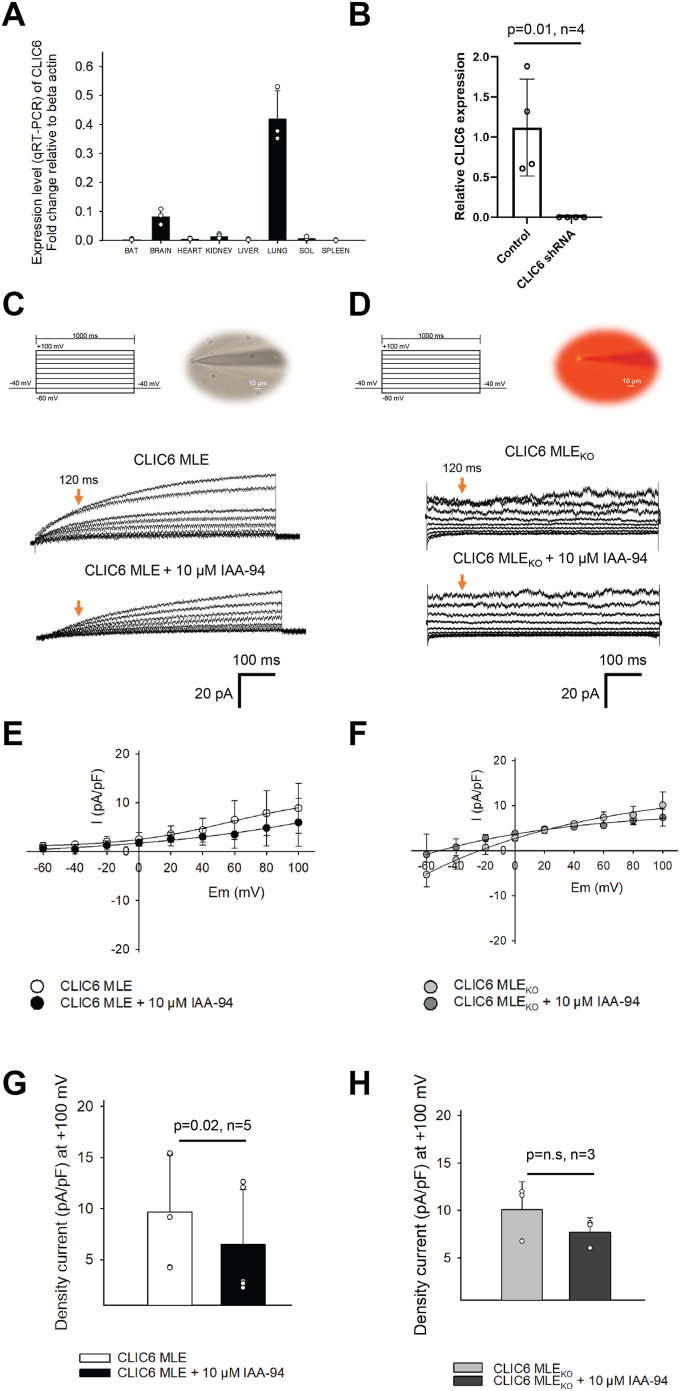
**Functional expression of CLIC6 in MLE cells.***A*, organ-specific mRNA expression of *clic6*. mRNA was isolated from brown fat (BAT), brain, heart, kidney, liver, lung, soleus muscle, and spleen of 2 months old mice. Representative graphs of relative expression, and quantification of *clic6* mRNA. Expression of *clic6* was normalized to beta-actin (housekeeping gene) where the expression was found to be highest in the lungs followed by the brain, kidney, and soleus muscle. *B*, RNA was isolated from MLE cells either untreated or treated for 48 h with CLIC6shRNA. Expression of CLIC6 was analyzed by qPCR. We did not detect any significant signal in MLE cells treated with CLIC6 shRNA. *C*, voltage-step protocol used for MLE cells and MLE cell (bar scale:10 μm) attached to the patch pipette for recording Cl^−^ currents. Representative trace of CLIC6 current recording in MLE cells before (*top panel*) and after adding 10 μM of IAA-94 (*bottom panel*). *D*, voltage-step protocol and MLE cell (attached to a patch pipette) transduced with lentivirus (*red* cell expressing the fluorescent reporter). The representative recordings of MLE cells (transduced with lentivirus) before (*top*) and after adding 10 μM of IAA-94 (*bottom*). The Cl^−^ channel kinetics were different from wildtype MLE cells. MLE cells lacking CLIC6 lacked the slow Cl^−^ kinetics component. *E*, current–voltage plot of the recordings presented in *panel C*. The *orange arrows* indicate the time point (120 ms) at which the current was measured for the I-V plot. The current was normalized to the MLE cell capacitance. *F*, current–voltage plot of the recordings presented in *panel D* for MLE cells lacking CLIC6. *G*, current density at +100 mV before and after the addition of 10 μM IAA-94 in MLE cells (*p* = 0.02, n = 5). *H*, current density at +100 mV before and after the addition of 10 μM IAA-94 in MLE cells transfected with lentivirus (p = n.s, n = 3). Error bars represent the mean ± standard deviation (SD), and significance was calculated by students’ *t* test (paired). CLIC, chloride intracellular channel; MLE, mouse lung epithelial.

CLIC6 was shown to be expressed in the choroid plexus, cerebellum, and other regions of the brain where it localizes with dopamine (D3) receptors (23, 24). We quantified the expression of CLIC6 in mRNA isolated from the major organs of C57BL6 mice. Organ-specific mRNA expression of CLIC6 in the mouse brain, heart, kidney, liver, lung, spleen, soleus muscle, and brown fat was analyzed by performing qRT-PCR, and beta-actin was used as an internal control. The results demonstrated that *clic6 mRNA* was highly expressed in the lung, moderate in the brain, and low in the heart, kidney, liver, spleen, soleus muscle, and brown fat (Fig. 6*A*). To determine whether there are IAA-94 sensitive currents present in lung cells, we recorded currents (Fig. 6) from MLE cells. MLE cells display robust expression of slow activation current which was blocked by IAA-94 (33 ± 10%, *p* = 0.02, n = 5, Fig. 6, *C*, *E*, and *G*), indicating that there are additional Cl^−^ currents present in lung epithelial cells (47). We also used lentivirus expressing CLIC6 shRNA and RFP reporter (Fig. 6, *B*, *D*, *F*, and *H*) to ablate CLIC6 from MLE cells, and the relative CLIC6 expression after 48 h transduction was corroborated by qPCR (Figs. 6*B* and S5). Cl^−^ currents were recorded from cells expressing RFP as they lack CLIC6. As shown in Figure 6, *D*, *F*, and *H*, MLE cells lacking CLIC6 showed a Cl^−^ current that was not sensitive to IAA-94. The Cl^−^ current showed a fast activation but without any effect by IAA-94 (Fig. 6, *D*, *F*, *and H*). In lung epithelial cells, other chloride channels (ClC-2) and outward rectifier chloride channel were shown to be active (47), but they do not show slow activation as seen for MLE cells and are not sensitive to IAA-94 (47). These results indicate that CLIC6 is present and functional in MLE cells.

## Discussion

Parchorin was first discovered in 1999 in rabbit gastric parietal cells (26, 48). It was described as a phosphorylated protein with a molecular weight of 120 kDa and named as pp120 (48). The pp120 was found to be present in abundance in tissues associated with water transport including the parietal cells and the choroid plexus and thus named parchorin. It was found to be present in the cytosol with a part of the protein translocating to the apical membrane in activated parietal cells without any change in the state of phosphorylation (48). The pp120 showed sequence homology to CLICs, and it was placed among the CLIC family as CLIC6. Parchorin was named CLIC6 and found to be distributed exclusively in tissues that are involved in transepithelial secretion such as saliva, tears, aqueous humor, cerebrospinal fluid, urine, periciliary tracheal fluid, and gastric juice. CLIC6 was mapped to 21q22.12, and it was distinct from other CLICs in terms of longer N terminus and GC-rich segment which encodes a 10 amino acid motif (AEGPAGDSVD) repeated 14 times (23).

Similar to other CLIC family members, CLIC6 is also present in the dimorphic state, *i.e.*, soluble and membrane forms (5). However, on removal of Cl^−^ from the extracellular medium, GFP-CLIC6 translocates to the membrane in LLC-PK1 cells, implicating a possible Cl^−^-dependent mechanism in membrane translocation (26). On overexpression of GFP-CLIC6, Cl^−^ efflux was only found to be twice as compared to untransfected cells which could be due to the presence of a native CLIC6 in LLC-PK1 cells (26). There were valid concerns about the possibility of CLIC6 as an ion channel, or an activator, or a regulator of a Cl^−^ channel. These concerns were augmented by the inability to measure currents in oocytes or transepithelial electrical resistance of Madin-Darby canine kidney (MDCK) cells transfected with CLIC6 (23). In the same study, it was shown that CLIC6 localizes to the cytosol and perinuclear spaces but not the plasma membrane in MDCK cells (23), thus explaining the lack of CLIC6-mediated currents in MDCK cells. We addressed this highly significant concern by ectopically expressing CLIC6 in HEK-293 cells in the current study and corroborated their expression in the plasma membrane. The MDCK cells need basolateral polarization, and it depends on cell–cell contacts. Lack of polarity disrupts the distribution and localization of proteins (49). In an earlier study, an anion channel of 460 pS conductance was discovered in the apical surface, and 46 pS conductance Cl^−^ channel was found in the basolateral cell membrane of MDCK cells (50). However, in subconfluent MDCK cells, no spontaneous anion channel activity was detected (50), supporting the fact that the absence of CLIC6 in earlier studies (23) could be attributed to the lack of polarity of MDCK cells used for studies.

The CLIC6 is the largest protein among all the CLICs, and similar to other CLICs, it has one predicted transmembrane domain (in between 487–512 amino acids, Fig. S4). To form a functional ion channel, CLIC6 will need to oligomerize and insert into the lipid bilayer. A functional CLIC1 is predicted to have a minimum of four monomers (35, 51). Lipids play a major role in the insertion of CLICs into the bilayers (35, 45), and recent studies have shown that divalent cations can also facilitate their insertion into the membranes. We have discovered that CLIC6 forms a functional channel with a conductance ∼3 pS and a rectification at positive holding potentials. The rectification is unique to CLIC6 and is not observed for other CLIC family members (5). The recording solution contains Cl^−^ and is nearly symmetrical (130 mM *versus* 135 mM) which could result in possible interaction of Cl^−^ ions with a binding site near the pore region of the channel. The binding of permeant ions to the obligatory binding site is known to control the rate of movement of ions through the channel and is also observed for BK channels (52). Further, Cl^−^ levels in recording solutions can also saturate the ion flux as the intracellular Cl^−^ concentration increases above the physiological level. We also tested whether IAA-94 can block the CLIC6 activity as IAA-94 is known to block the majority of CLIC family members (5). IAA-94 blocked the CLIC6 activity at the positive holding potential and ablated the rectification component of the channel. The tail currents were reduced by IAA-94. These results indicate that CLIC6 is highly active at positive holding potentials. The transmembrane domain of CLIC1 and CLIC6 are 100% conserved (Fig. S4), but the biophysical properties of channels are distinct. The difference in channel kinetics in CLIC6 *versus* other CLICs could arise from two major factors: (1) the longer N terminus of CLIC6 and (2) specific interactors of CLIC6 such as kinases as reported earlier (26). In HEK-293 cells transfected by CLIC6, the addition of DTT in the bath solution modulated that channel activity. The effect of DTT was obliterated when the cysteine residue located on top of the transmembrane domain was replaced by alanine indicating that the cysteine residue is also facing the extracellular side as reported for CLIC1 (35).

Chloride channels are known for their anion selectivity (5, 16). We first tested cation *versus* anion selectivity in KCl *versus* KMeSO_4_ solutions in CLIC6-transfected HEK-293 cells. In the presence of K^+^ ions, the macroscopic currents of CLIC6 were different from the ones recorded under NMDG-Cl^−^. This could be attributed to the activation of endogenous K^+^ channels in HEK-293 cells or different cations that can affect the anion mobility (53) and affect the binding of anions to the selectivity site in anion channels (54). In addition, CLICs are known to form poorly selective ion channels (35), and the presence of a smaller cation could allow them to pass through but at a lower rate which could also affect the macroscopic currents. Among different anions, maximum macroscopic currents were observed for chloride followed by bromide and fluoride ions. These results along with selectivity data (5, 35, 36, 37) for other CLICs indicate that CLICs are anion channels with a range of preferences for different anions. Among all the CLIC proteins, CLIC6 is highly selective for Cl^−^ ions. Since all the residues in the transmembrane domain are conserved among CLIC proteins, the selectivity and preference for Cl^−^ ions could arise from other parts of the proteins or various protein interactors.

One of the major cellular regulators of Cl^−^ channels is cellular pH. Low pH has been implicated in conformational changes in CLIC proteins. For CLIC1, acidic pH affects its insertion, stability, folding, and increased activity (44, 55, 56, 57). In whole-cell recordings, pH was maintained at pH 7.2, and when the same cell was perfused with pH 6.2, we observed a slight reduction in the macroscopic currents. At pH 7.2, IAA-94 reduced macroscopic currents, but surprisingly at pH 6.2, we did not notice any IAA-94-mediated block. There is a possibility at low pH the channel configuration is different from pH 7.2. For CLIC1, global conformation is not altered within the range of pH 5.5 to 8.2, but at the low pH, intermediate states were observed. The alpha helix 1 which is a major component of the transmembrane domain undergoes an unfolding and is destabilized at low pH. The absence of IAA-94 mediated block, and decreased macroscopic currents for CLIC6 support change in the configuration of the channel structure. Further, we discovered a histidine residue (H648) which is conserved across all the CLICs and decreases the macroscopic currents and ablates IAA-94-mediated block of CLIC6 currents which was similar to low pH. We predict that the residue H648 is also a pH sensor in other CLIC proteins. There are possibilities of the presence of other Cl^−^ channels in HEK-293 cells (58, 59) which can get activated at lower pH and are not sensitive to IAA-94, but CLIC6 specifically shows a reduction in the current density.

In earlier studies, in addition to divalent cations (60), DTT was implicated in membrane insertion of CLIC proteins (35, 46, 61). CLIC1, CLIC4, and CLIC5 readily insert in the presence of DTT. However, a recent study showed that oxidation promotes the insertion of CLIC6 into membranes (25). In HEK-293 cells transfected with CLIC6, we recorded currents in solutions devoid of DTT or H_2_O_2_. In whole-cell configuration, when DTT is added to the bath solution, we observed an increase in the macroscopic currents. In contrast, H_2_O_2_ showed no change in the macroscopic currents. In a recent study, addition of 2 mM H_2_O_2_ alone failed to induce any changes in CLIC6 (25). However, H_2_O_2_ along with large unilamellar vesicles containing CLIC6 facilitated the formation of discrete high-order oligomers of CLIC6 which implies that oxidative conditions and the presence of lipids are vital for the oligomerization of CLIC6 (25). Once the channel is formed, H_2_O_2_ does not affect its function.

These results indicate that oxidation has no impact once the channel is inserted in the membrane (62), although it might facilitate its insertion into the membranes. Since we observed an increase in macroscopic currents in the presence of DTT which was similar to CLIC1 and CLIC5, we focused on cysteine residues involved in redox regulation. The cysteine residue at 487 corresponds to cysteine 24 in CLIC1 which is conserved across all the CLIC proteins including *Dm*CLIC (5). When CLIC6 C487 was replaced with alanine, we noticed a 22.7% reduction in peak current at +100 mV. Further, DTT failed to increase the macroscopic currents for CLIC6 C487A. These results for the first time showed that CLIC6 is a redox-regulated channel, and the structure-function approach also indicates that the current flow is through CLIC6 and not another associated Cl^−^ channel.

In MLE cells, we recorded IAA-94-sensitive Cl^−^ currents which were not present in MLE cells transduced with CLIC6 shRNA. The channel kinetics were different from CLIC6-transfected HEK-293 cells which indicates either a presence of other modulatory proteins or a different configuration of CLIC6. Nevertheless, these results indicate that CLIC6-like, IAA-94 sensitive channels are present in MLE cells. The functional role of these Cl^−^ channels is not yet established. Given CLIC1 is a key component of a Cl^−^ channel complex involving CFTR (63), we anticipate CLIC6 to be a part of a Cl^−^ channel complex in lung cells.

**Figure 7:**
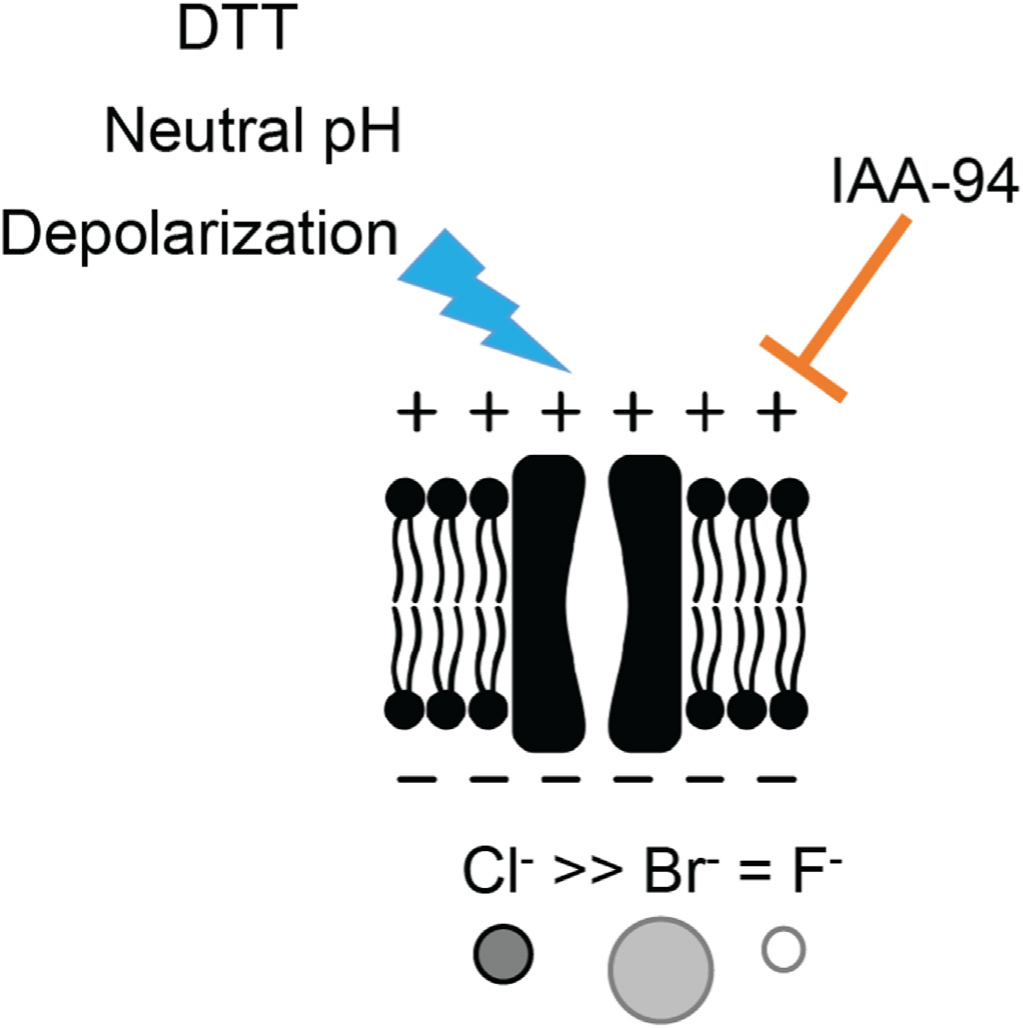
**Model of CLIC6 in the cell membrane and its regulation.** Our results indicate that CLIC6 forms a Cl^−^-selective channel in the cell membrane. The activity of CLIC6 is blocked by IAA-94. The channel can be activated by membrane depolarization, neutral pH, and high redox potential (DTT). CLIC, chloride intracellular channel.

In summary, we have shown that CLIC6 forms a functional IAA-94-sensitive Cl^−^ channel which is regulated by DTT and pH (Fig. 7). Our structure-function approach also provides evidence for the role of a conserved N terminus cysteine residue in the redox-regulation of CLIC6 in the native cellular environment.

## Experimental procedures

### Molecular cloning

pcDNA3.1 encoded 3× Flag-tagged human CLIC6 (GenBank accession number: NM_001317009) procured from NovoPro was used as a template for site-directed mutagenesis by the “Quick change” method. Primers carrying (Table S1) cysteine at position 487 to alanine mutation were used to generate CLIC6 C487A mutant. The PCR reaction mixture was prepared according to the manufacturer’s protocol [Agilent (600850)]. The amplified PCR product was digested with *Dpn1* enzyme at 37 °C for 6 h and transformed into *E. coli DH5α* competent cells. The positive colonies were screened by Sanger sequencing. Plasmids were isolated using commercial kits (Qiagen).

### MLE cell culture and transduction

MLE-12 cells were cultured in Dulbecco's modified Eagle's medium:F12 medium containing 2.5% (*v*/*v*) FBS and 1% (*v*/*v*) penicillin-streptomycin. MLE cells were maintained at 37 °C in the incubator supplied with 5% (*v*/*v*) CO_2_. Once MLE cells reached 70% confluency, they were transduced with lentivirus (1 × 10^5^ transduction units, LipExoGen Biotech) containing human CLIC6 shRNA with RFP (RFP, BSD) reporter. After 48 h incubation, MLE cells were washed with sterile PBS buffer and trypsinized with 0.25% Trypsin–EDTA at room temperature. Cells were seeded onto coverslips and incubated with Dulbecco's modified Eagle's medium:F12 medium containing 2.5% (*v*/*v*) FBS and 1% (*v*/*v*) penicillin–streptomycin for 1 h before performing electrophysiology measurements.

### Immunochemistry

HEK-293 cells were transfected with pcDNA3.1 Flag-CLIC6 lipofectamine 3000 according to the manufacturer’s instructions. HEK-293 and MLE cells were incubated with wheat germ agglutinin (1:1000 dilution) on ice for 60 min. Cells were washed and fixed with 4% (*w*/*v*) paraformaldehyde. Cells were incubated with anti-Flag antibodies [1:500, Sigma Aldrich (F1804)] for 16 h. Cells were washed with PBS and incubated with secondary antibodies for 60 min at room temperature. After 60 min, cells were washed with PBS and incubated with DAPI (1:30,000). Cells were washed and mounted on slides using Mowiol (Sigma-Aldrich, 81381). Cells were imaged with Nikon A1R at 60× (1.44 NA). Images were postprocessed using a median filter (64) and image J.

### Western blot

HEK-293 cells transfected with pcDNA3.1 Flag-CLIC6 plasmid were lysed in RIPA buffer for 1 h at 4 °C with continuous shaking. After 1 h, samples were centrifuged at 12,000*g* for 20 min at 4 °C, the supernatant was collected and loaded on 4 to 20% (*w*/*v*) SDS–polyacrylamide gel electrophoresis and transferred to nitrocellulose membranes. Protein loading was corroborated with Ponceau S staining. Membranes were blocked with an LI-COR blocking buffer and washed with Tris-buffered saline (TBS) before incubating overnight with various primary antibodies at 4 °C. The primary antibody used was anti-FLAG [1:500, Sigma Aldrich (F1804)]. After washing with TTBS (20 mM Tris buffer saline containing 0.05% (*v*/*v*) Tween-20) for 10 min each, membranes were incubated with anti-mouse secondary antibody conjugated to IR800 (1:1000, Li-COR Biosciences 92532210) for 1 h at RT and washed again with TTBS for 5 min. Signals were visualized using an infrared fluorescence system (Bio-Rad).

### qRT-PCR

All organs were surgically excised from 2-month-old C57BL/6C mice. Total RNA was prepared using TRIZOL reagent (Invitrogen) as per the manufacturer’s instructions. This was followed by column-RNase-free DNAse digestion (Qiagen) and clean-up with RNeasy mini kit (Qiagen). 2 μg of RNA after clean-up was reverse transcribed in a 20 μl reaction volume with Omniscript Reverse Transcription kit (Qiagen) using Oligo dT primers. Real-time qPCR was performed using Power up SYBR green master mix in QuantStudio 3 (Applied Biosystems), 1 μl of RT reaction product, and 200 nM primer pairs (Table S1) in a 10 μl reaction volume, according to MIQE guidelines (15, 18, 64, 65). The thermal cycling conditions included an initial denaturation at 95 °C for 10 min and 40 cycles of 95 °C for 45 s, 60 °C for 1 min, and 72 °C for 45 s. The controls used were (−) RT (cDNA with no reverse transcriptase), and primers used to amplify beta-actin (Table S1). All samples were run in triplicates. The fold change in the expression of CLIC6 in the respective organs has been plotted relative to beta-actin.

### Electrophysiology

Ion channel currents were recorded with an EPC10 USB HEKA amplifier in the whole-cell configuration and a voltage-step protocol from −100 mV to +100 mV (for HEK-293 cells) and −60 mV to +100 mV (for MLE cells) in 20 mV increments with a holding potential of −40 mV and 1000 ms of duration. The tail current was generated at −40 mV after running the voltage-step protocol (Fig. 1*G*) and from −100 to +100 mV after running a depolarized step of +120 mV (Fig. S2). The tail current was measured at the peak current (1.025 s) before and after the addition of IAA-94. The single-channel activity was recorded in cell-attached mode on a gap-free protocol for 100 s. The signal was digitally filtered at 800 Hz in whole-cell and 20 Hz in single-channel recordings. Patch-clamp pipettes had a resistance between 5 and 7 MΩ, and the intracellular (pipette) solution composition in [mM] was 130 NMDG-Cl, 1 CaCl_2_, 1 MgCl_2_, 10 glucose, 5 EGTA, and 10 Hepes (pH 7.2), and the extracellular (bath) solution was 135 NMDG-Cl/Br/F, 1 CaCl_2_, 10 glucose, and 10 Hepes (pH 7.2 and 6.2). Osmolarity was measured by Fiske 210 Micro-Osmometer. Data were analyzed using PatchMaster v2x73 software.

### Automated electrophysiology measurements

HEK-293 cells transfected with pcDNA3.1 CLIC6 were used 48 h after transfection. The currents were measured by patch-clamping in the whole-cell configuration. The experiments were conducted with a high-throughput automated patch-clamp system SyncroPatch 384i (Nanion) and its customized recording chip (NPC-384T 1 X L-Type, Nanion). Chips made up of thin glass containing one hole per well with low resistance (1–6 MΩ) were used for HEK-293 cell lines. Pulse generation and data collection for HEK-293 cells were performed with PatchController384 V1.4.1 and DataController384 V1.3.3 according to Nanion’s standard operating protocol. The intracellular solution contained (in mM) 207 NMDG, 30 HCl, 10 EGTA, 1 CaCl_2_, 10 Hepes, and 1 D-glucose, and the pH was balanced to 7.2 with sulfuric acid. The extracellular solution contained (in mM) 130 NaCl, 4 KCl, 10 BaCl_2_, 5 D-glucose, and 10 Hepes (pH 7.2). All cells were recorded simultaneously. After initiating the experiment in HEK-293 cells, cell catching, sealing, whole-cell formation, liquid application, and data acquisition were performed sequentially according to Nanion’s instructions. We successfully obtained giga-ohm seals in 91 cells in recording solutions. The current-voltage relationships of the chloride currents were recorded by holding the resting membrane potential at −40 mV and stepping from −100 mV to +100 mV in a 20 mV interval. Each step was held for 1s duration. The currents obtained were sampled at 20 kHz. The SyncroPatch 384i (Nanion Technologies) platforms have a software package for data acquisition (PatchControl 384) and for data analysis (DataControl 384) which were used to characterize CLIC6 currents.

### Statistical analysis

A minimum of three independent experiments were carried out. All the data were analyzed by Sigma plot and Origin. Error bars represent the mean ± standard deviation (SD). Significance was calculated by the student’s *t* test (paired) and ANOVA (1-way).

## Data availability

All data generated and analyzed during this study are available from the corresponding author upon reasonable request.

## Supporting information

This article contains supporting information.

## Conflict of interest

The authors declare that they have no conflicts of interest with the contents of this article.
