# Supplementary material for: Biophysical characterization of chloride intracellular channel 6 (CLIC6)
Source: J Biol Chem. 2023 Oct 12;299(11):105349. doi: 10.1016/j.jbc.2023.105349 (PMC10641671; doi:10.1016/j.jbc.2023.105349)
Supplement: Supporting Information [file mmc1.pdf]

Supporting information

Biophysical Characterization of Chloride Intracellular Channel 6 (CLIC6)

Veronica Loyo-Celis[1], Devendra Patel[1], Shridhar Sanghvi[1,2], Kamalpreet Kaur[1], Devasena Ponnalagu[1,3], Yang Zheng[1], Sahej Bindra[1], Harmeet Rireika Bhachu[1], Isabelle Deschenes[1], Shubha Gururaja Rao[4] & Harpreet Singh[1,2*].

[1]Department of Physiology and Cell Biology, College of Medicine, The Ohio State University, Columbus, OH

[2]Department of Molecular Cellular and Developmental Biology, The Ohio State University, Columbus, OH

[3]Department of Pharmacology, The University of Washington, Seattle, WA

[4]Raabe College of Pharmacy, Ohio Northern University, Ada, OH

***Correspondence to:**

Harpreet Singh Ph.D.
Department of Physiology and Cell Biology,
333W 10th Avenue
The Ohio State University College of Medicine
Columbus, OH, 43210, USA
Harpreet.singh@osumc.edu

Running title: Biophysical Characterization of CLIC6

Keywords: chloride channel; redox-regulation; pH-regulation; IAA-94; anion transport

Supplementary table 1: Sequence of primers. Primers used for qPCR (CLIC6 and Beta-actin) are listed in the table. The primer set used for replacing the N-terminus cysteine residue and the C-terminus histidine residue to alanine is provided in the table.

Name	Forward Primer (5'-3')	Reverse primer (5'-3')
CLIC6	CAC GAC ATC ACC CTC TTT GT	CCC TTT AGAC CAG AGG ATC ATA AA
Beta-actin	TAT GCC AAC ACA GTG CTG TCT GG	TAC TCC TGC TTG CTG ATC CAC AT
CLIC6 C487A	ATC GGA AAT GCC CCG TTT TCT CAG	CTG AGA AAA CGG GGC ATT TCC GAT
CLIC6 H648A	CTT ACC AAG CTC GCT ATT ATT AAG ATT GTG GCC	GGC CAC AAT CTT AAT AAT AGC GAG CTT GGT AAG

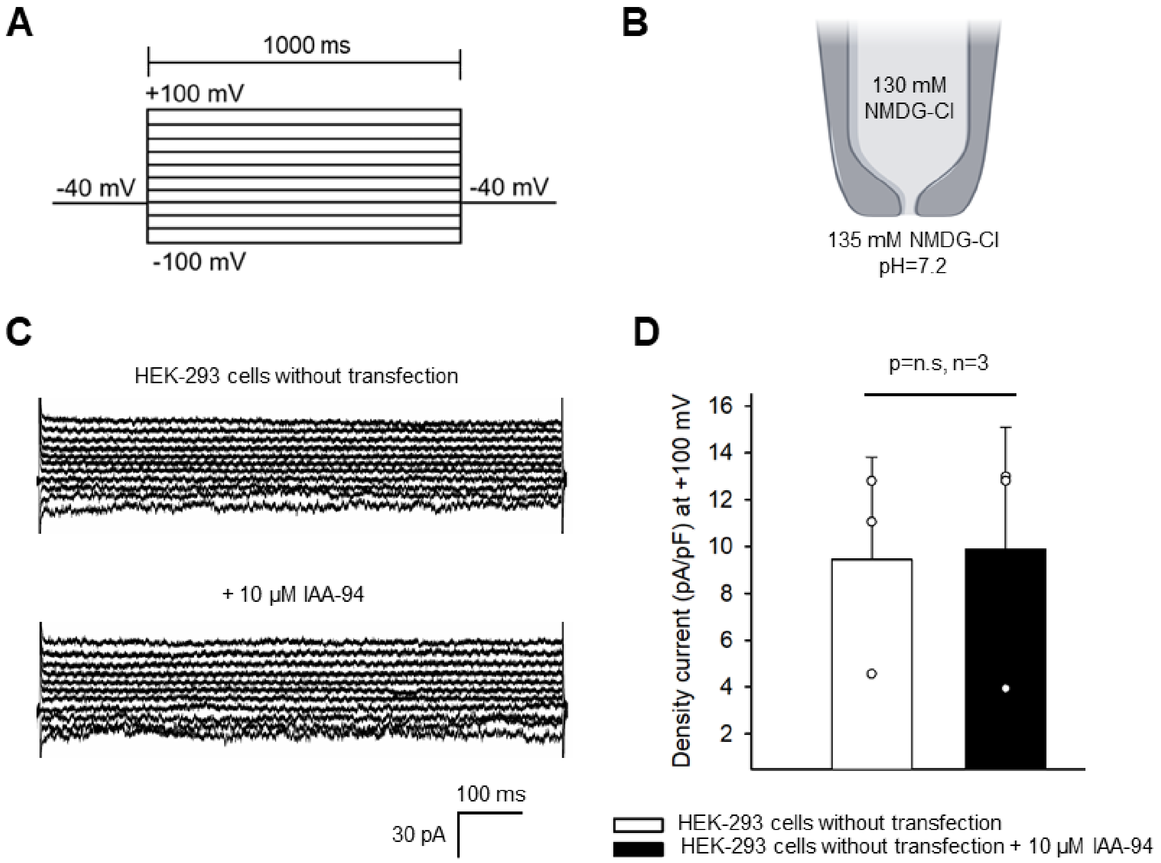

Supplementary Figure 1. The IAA-94 current block effect was abolished in HEK-293 cells without transfection. A. Voltage-step protocol. **B.** Buffer composition for pipette and bath solutions. **C.** Whole-cell recordings in HEK-293 cells without transfection before (up) and after the addition of 10 μM IAA-94 (down). **D.** Density current (pA/pF) at +100 mV from **C** (p=n.s, n=3). There was no effect of IAA-94 on Cl^- currents recorded from untransfected HEK-293 cells. Error bars represent the mean ± standard deviation (SD).

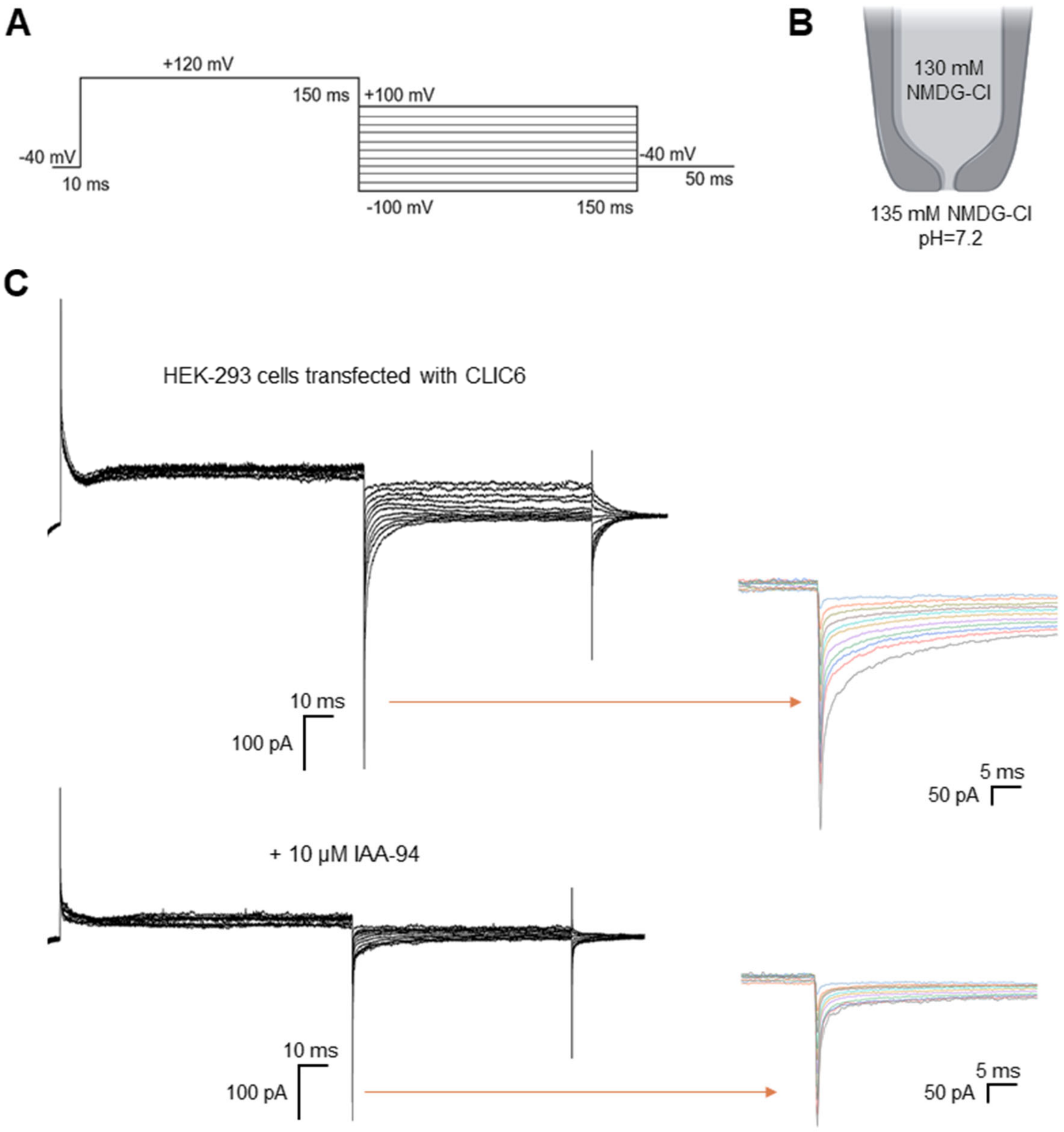

Supplementary Figure 2. Tail currents of CLIC6. A. Voltage protocol for obtaining tail currents. **B.** Composition of pipette and bath solutions. **C.** Tail current recordings generated in HEK-293 cells transfected with CLIC6 before (upper) and after (down) addition of 10 μM IAA-94. Orange arrows indicate zoom region of tails (colors represent distinct holding potentials).

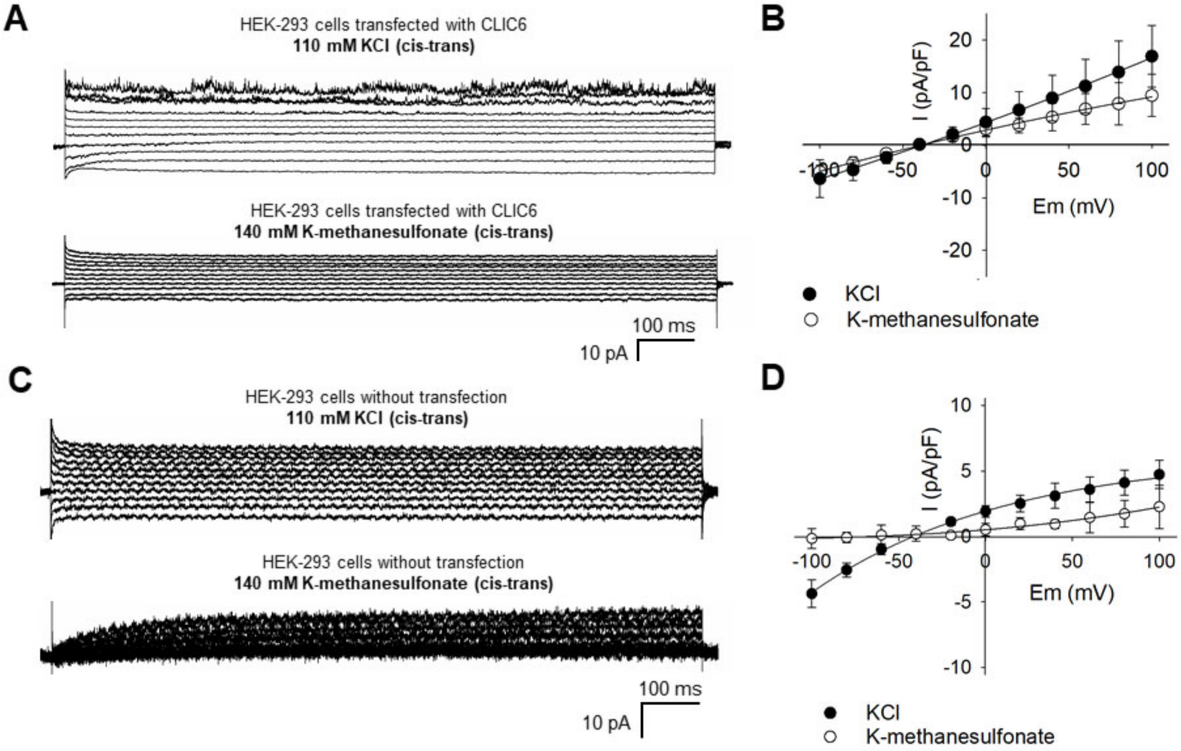

Supplementary Figure 3. Permeability of CLIC6 in potassium solutions. A. Representative traces of CLIC6 expressed in HEK-293 cells recording in (110:110:cis:trans mM) KCl (top) and (140:140:cis:trans mM) $KMeSO_4$ (bottom). **B.** Current-voltage graph of recordings showing in **A**. (n=3 for KCl condition and n=4 for $KMeSO_4$). **C.** Representative traces of HEK-293 cells without transfection recording in (110:110:cis:trans mM) KCl (top) and (140:140:cis:trans mM) $KMeSO_4$ (bottom). **D.** Current-voltage graph of recordings showing in **C**. The current was measured at 900 ms, and normalized for HEK-293 cells capacitance (n=5). In HEK-293 cells transfected with CLIC6 the E_r is -40 mV, the same as we shown in (**Fig. 1*D***) implying that Cl^- ions are moving through the CLIC6. The Cl^- concentration in recordings showing in (**Fig. 1*C***) was almost the same (130:135 cis:trans mM) than in the recordings showing in (**Supplementary Figure 3*A***) (110:110 cis:trans mM), however the counter cation was different because we replace NMDG-Cl for KCl. The lower permeability rate and ion channel currents could also arise from competition between K^+ and Cl^- ions.

Putative transmembrane domain

```
471 IKKYLRAGYDGESIGNCPFSQRLFMILWLKGVIFNVTTVDLKRKPADLQNLAPG CLIC6
  8 VELFVKAGSDGAKIGNCPFSQRLFMVLWLKGVTFNVTTVDTKRRTETVQKLCPG CLIC1

641 CNLLPKLHIIKIVAKKYRDFEFP CLIC6
178 CNLLPKLHIVQVVCKKYRGFTIP CLIC1
```

Supplementary Figure 4. Sequence alignment of N-terminus and putative transmembrane domain of human CLIC6 (top, NP_001303938.1) and human CLIC1 (bottom, UQL51164.1). Conserved residues are highlighted in orange color. Putative transmembrane domain is highlighted by yellow. Conserved cysteine and histidine residue in CLIC6 (C487 and H648) and CLIC1 (C24 and H185) are highlighted by blue. C487 and H648 of CLIC6 was mutated to alanine residue.

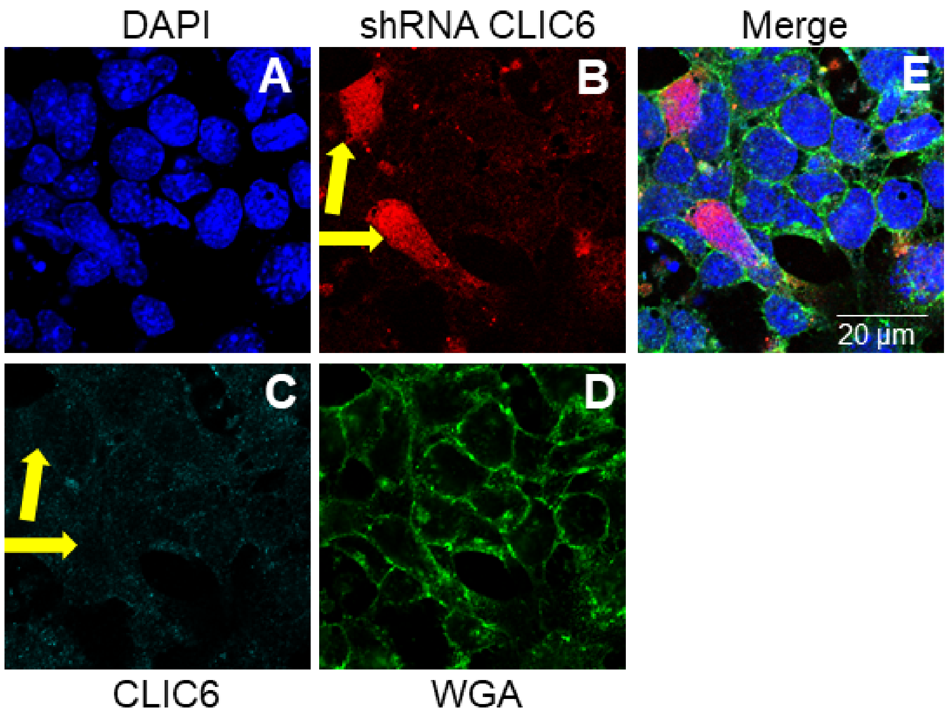

Supplementary Figure 5. CLIC6 expression down regulated by CLIC6 shRNA. A. MLE cells labeled with DAPI. **B**. MLE cells transduced with lentivirus containing CLIC6 shRNA. Red colored cells highlighted by yellow arrows indicate MLE cells transduced with CLIC6 shRNA. **C.** MLE cells incubated with anti-CLIC6 antibody. Yellow arrows from C indicate down regulation of CLIC6 in cells transduced with CLIC6 shRNA. **D.** MLE cells labelled with WGA to label plasma membrane. **E.** Merge image of A, B. C and D. Scale bar: 20 µm
